# Efficacy of *Lamium album* as a natural fungicide: impact on seed germination, ergosterol, and mycotoxins in *Fusarium culmorum*-infected wheat seedlings

**DOI:** 10.3389/fmicb.2024.1363204

**Published:** 2024-02-23

**Authors:** Pascaline Aimee Uwineza, Monika Urbaniak, Łukasz Stępień, Anna Gramza-Michałowska, Agnieszka Waśkiewicz

**Affiliations:** ^1^Department of Chemistry, Poznan University of Life Sciences, Poznan, Poland; ^2^Plant-Pathogen Interaction Team, Institute of Plant Genetics, Polish Academy of Sciences, Poznan, Poland; ^3^Department of Gastronomy Science and Functional Foods, Poznan University of Life Sciences, Poznan, Poland

**Keywords:** *Triticum* genus, plant extract, *Lamiaceae*, PCR, trichothecenes, zearalenone

## Abstract

*Fusarium culmorum* is a major wheat pathogen, and its secondary metabolites (mycotoxins) cause damage to plants, animals, and human health. In the era of sustainable agriculture, eco-friendly methods of prevention and control are constantly needed. The use of plant extracts as biocontrol agents has gained popularity as they are a source of active substances that play a crucial role in fighting against phytopathogens. This study evaluated the impact of *Lamium album* on wheat seed germination and seedling growth. In a pot experiment, the effect of *L. album* on wheat seedlings artificially inoculated with *F. culmorum* was evaluated by measuring seedling growth parameters, and by using chromatographic methods, ergosterol and mycotoxins levels were analyzed. The results showed that the phytotoxic effect of *L. album* flower extracts on wheat seed germination and seedling growth was concentration dependent. The radicle length was also reduced compared to the control; however, *L. album* did not significantly affect the dry weight of the radicle. A slight phytotoxic effect on seed germination was observed, but antifungal effects on artificially infected wheat seedlings were also confirmed with the reduction of ergosterol level and mycotoxins accumulation in the roots and leaves after 21 days of inoculation. *F. culmorum* DNA was identified in the control samples only. Overall, this study is a successful in planta study showing *L. album* flower extract protection of wheat against the pathogen responsible for Fusarium crown and root rot. Further research is essential to study the effects of *L. album* extracts on key regulatory genes for mycotoxin biosynthetic pathways.

## 1 Introduction

Wheat is a crucial cereal crop in the *Triticum* genus, and it serves as a dietary staple for nearly 40% of the world's population (Iqbal et al., 2022). Despite being the second most crucial staple after rice (Singh et al., 2016; Özdemir, 2022), *Fusarium* infections in wheat present a global challenge, causing reduced yield, compromised quality, and the accumulation of harmful mycotoxins (Bota et al., 2021; Riaz Ejaz et al., 2023). *Fusarium culmorum*, a soil-borne fungus (Antalová et al., 2020), is a primary wheat pathogen, with *Fusarium* root rot, *Fusarium* crown rot, and *Fusarium* head blight (FHB) being particularly threatening due to mycotoxin contamination (Bottalico and Perrone, 2002; Scherm et al., 2013; Antalová et al., 2020). Zearalenone (ZEN) and type B trichothecenes, such as deoxynivalenol (DON), acetyl-deoxynivalenol (3-ADON and 15-ADON), and nivalenol (NIV) (Alisaac et al., 2023) pose severe risks to human health, food security, and economy (Ostry et al., 2017). Therefore, managing *Fusarium* diseases in cereals, especially wheat, is crucial for ensuring food security and safeguarding human health.

Efforts to combat *Fusarium* infections in wheat have involved substantial investments in genetic resistance, crop rotation, and other practices (Özdemir, 2022). However, complete host resistance remains elusive (Wagacha and Muthomi, 2007), underscoring the ongoing importance of fungicides (Zubrod et al., 2019). Synthetic fungicides, while effective, pose risks to human health and the environment (Castro et al., 2020; Seepe et al., 2021), forcing the exploration of natural alternatives, which are vital for sustainable crop cultivation. Plant extracts are recognized as safe and offer a promising solution (Abdallah et al., 2018), containing a blend of compounds, including phenolic acids, flavonoids, tannins, terpenes, and alkaloids, that can collaborate to inhibit the growth of phytopathogenic fungi (Deresa and Diriba, 2023). Moreover, plant extracts are highly effective against a wide range of pests and diseases, are relatively easy and cheap to produce, and show low toxicity against non-target organisms, including humans (Suteu et al., 2020). Significantly, these compounds not only limit fungal growth by inhibiting ergosterol biosynthesis as an indicator of fungal biomass (Perkowski et al., 2008) but also inhibit mycotoxin biosynthesis and trigger plant defense responses (Acheuk et al., 2022). However, to monitor the *in vivo* antifungal properties of a plant extract, it is essential to evaluate the phytotoxic effects on the target plant that requires protection, considering that certain studies have indicated their potential adverse effects.

*L. album* is a medicinal plant distributed in Europe, Asia, and Africa (Chipeva et al., 2013). To understand the mechanisms of action, researchers identified its chemical constituents and found that it has various chemicals, including iridoid glycosides, phenolic acids, flavonoids, alkaloids, triterpenes, and other compounds with diverse biological properties (Damtoft, 1992; Pereira et al., 2012; Pourmirzaee et al., 2019; Sulborska et al., 2020; Uwineza et al., 2021). In recent *in vitro* research*, L. album* flower extracts demonstrated promising effects against *Fusarium* species, emphasizing its versatile applications (Uwineza et al., 2023). Most studies focus on plant extracts' fungicidal or fungistatic effects (García-Ramírez et al., 2023). However, they often overlook the impact of these extracts on mycotoxin production by pathogenic fungi and phytotoxic effects on the crops. Furthermore, most of these studies are conducted *in vitro* (Kursa et al., 2022; Uwineza et al., 2022), while *in vivo* experiments are necessary to confirm the biocontrol effects of such plant extracts, as the data available is limited. In addition, for the plant extract to be effective, accurate identification of the pathogen is crucial. Identifying pathogens based on observed symptoms can be challenging, especially at the early stages of disease. Therefore, molecular methods, particularly those based on polymerase chain reaction (PCR), offer superior detection capabilities. The technique is a rapid and sensitive tool for detecting and identifying targeted DNA molecules of pathogens in plant tissues (Pszczółkowska et al., 2013).

The study aimed to assess the efficacy of *L. album* flower extract [obtained by using supercritical fluid carbon dioxide (SC-CO_2_)] on wheat seed germination and analyze its antifungal action against *F. culmorum* in artificially infected wheat seedlings. Our previous research has shown a promising *in vitro* effect of *L. album* against *F. culmorum* and *F. proliferatum* (Uwineza et al., 2023), and the literature has indicated that infections in seedlings and basal stems often originate from seed or soil-borne inoculum, leading to significant yield losses due to damaged seedlings, pre-harvest lodging, and impaired grain filling (Gebremariam et al., 2020). Therefore, controlling *Fusarium* infection from the early stage may promise better harvesting.

## 2 Results

### 2.1 Effect of *Lamium album* on wheat seed germination and seedling growth

The effect of *L. album* flower extract on wheat seeds was evaluated after 7 days of incubation. The results showed that *L. album* extracts inhibited wheat seed germination in a concentration-dependent manner (Table 1). The germination percentage was calculated using the Equation 1, where the control sample had a 100% germination percentage, while the treated samples showed a significant difference with a 65% germination percentage for 5% of extracts, while for 10% of extracts, the germination percentage was 45%. Additionally with the Equation 2, *L. album* flower extract reduced the seedling length vigor index from 669.50 to 303.86 compared to the control (2,860.00). It also decreased the seedling weight vigor index from 2.83 to 1.84 compared to the control value of 3.92 obtained using the Equation 3.

**Table 1:** Comparative effects of *L. album* on wheat seed germination.

**Sample name**	**Germination percentage [%]**	**Seedling length vigor index**	**Seedling weight vigor index**
Control	100^a^	2,860.00^a^	3.92^a^
5% extract	65^b^	669.50^b^	2.83^ab^
10% extract	45^c^	303.86^c^	1.84^b^

Values with different letters are statistically different (values obtained from the mean results of the treatment, α = 0.05).

**Figure 1:**
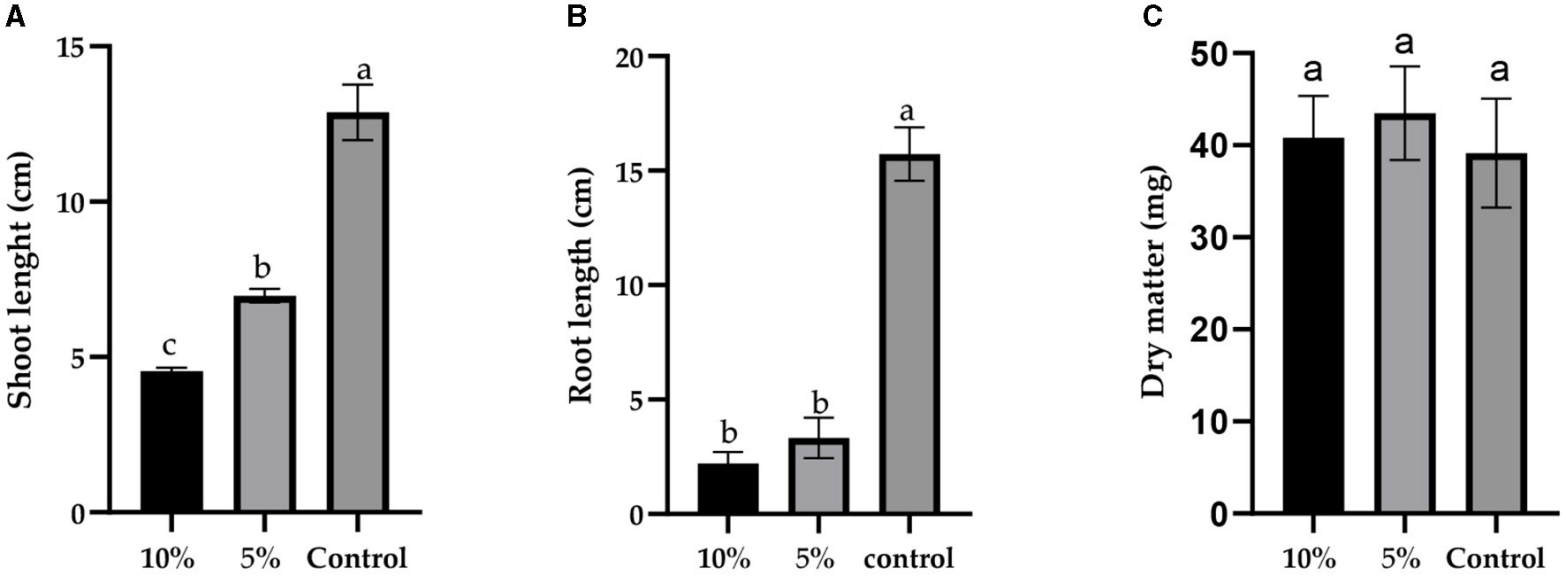
The effect of different concentrations of *L. album* extract (5 and 10%) compared to the untreated control: **(A)** the shoot length (cm), **(B)** root length (cm), and **(C)** dry matter (mg) of seedlings after 7 days of growth. Values with different letters are statistically different (mean ± standard deviation, α = 0.05).

**Figure 2:**
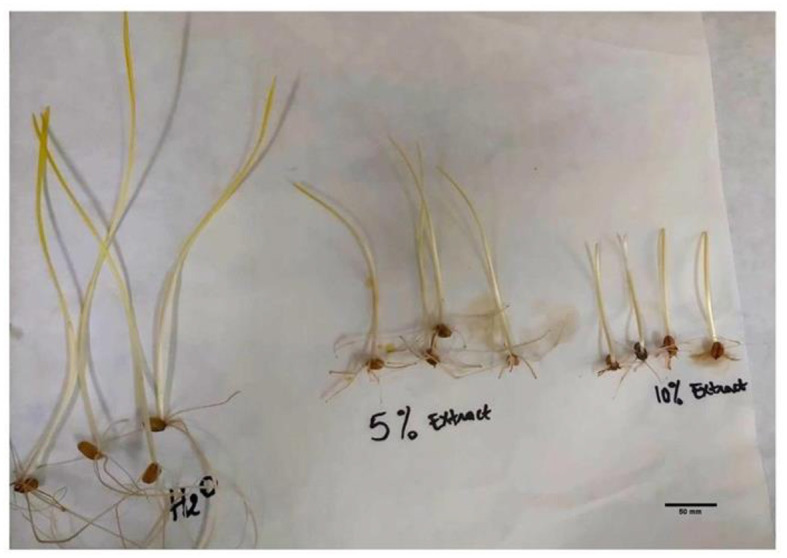
The effects of different concentrations of *L. album* flower extract (5% and 10%) compared to the untreated control (treated with water) on seedlings' shoot and root length after 7 days of incubation.

Furthermore, the concentration of *L. album* significantly influenced both shoot length (*F* = 259.3, *p* < 0.0001) and root length (*F* = 282.5, *p* < 0.0001) of wheat seedlings, as depicted in Figures 1, 2. However, there was no significant difference in dry matter (*F* = 0.7018, *p* = 0.5209). With an increase in *L. album* concentration, there was a noticeable decrease in shoot and root length.

In specific instances, such as at a 10% *L. album* concentration, the germinated seeds resulted in shoot and root lengths of 4.55 and 2.20 cm, respectively. In contrast, at a 5% *L. album* concentration, the shoot and root lengths were 6.975 and 3.32 cm, respectively, both shorter than the untreated control with shoot and root lengths of 12.88 cm and 15.73 cm, respectively.

### 2.2 Comparative effects of *L. album* extract on the physiological growth parameters of wheat seedlings in the presence and absence of *Fusarium culmorum* infection

**Figure 3:**
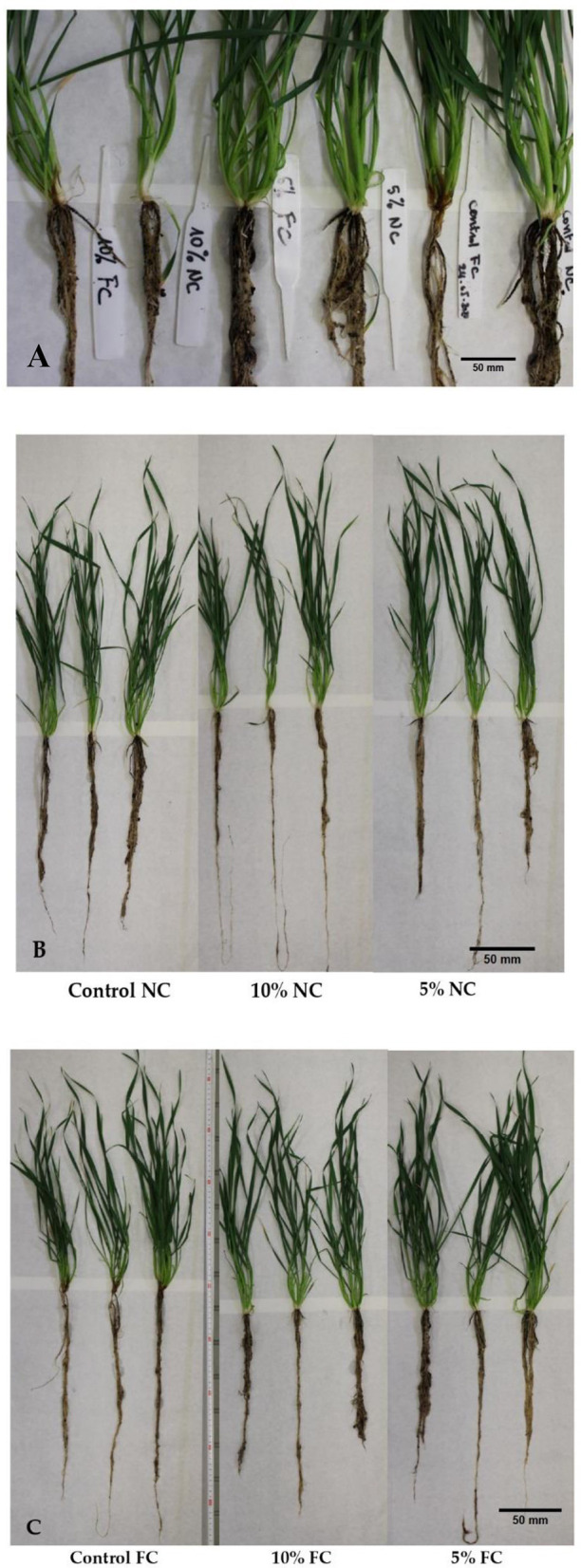
Differences in root phenotype among the harvested wheat seedlings after 21 days of infection: **(A)** comparative observation of the infected and non-infected seedlings, **(B)** non-infected seedlings, and **(C)** infected seedlings.

Physiological growth parameters of wheat seedlings were variably influenced by *L. album* flower extract and *F. culmorum* infection. With the data collected after 21 days of artificial infection of wheat seedlings, there are slight differences between the seedling root weight of the pathogen-inoculated control (Control_FC) and the inoculated and treated with 5% and 10% of *L. album* extract (5% _FC and 10% _FC) with a mass drop of approximately (−0.3 g). However, root weight loss was observed in non-infected seedlings treated with *L. album* extract (5% and 10% NC), with a mass drop of (−1.8 g) compared to the control. Similar effects were also observed in root length, except for non-infected seedlings treated with 10% of extracts (10% NC), which showed the longest roots (Table 2, Figure 3). The samples treated with 10% extract showed a stunting effect of *F. culmorum* on seedling root development, where the infected seedlings (10% _FC) showed a decrease in root length compared to non-infected seedlings (10% NC) but increased root weight. This reduction may signify a positive impact of *L. album* flower extract in combating *Fusarium*. Shorter roots could be a defense response, limiting *Fusarium* invasion and strengthening plant resistance. This observation underscores the potential antifungal efficacy of *L. album* extract.

**Table 2:** Effect of *L. album* extracts on the growth of infected and non-infected wheat seedlings.

**Sample treatments**	**Fresh weight [g]**	**Shoot length [cm]**	**Root length [cm]**	**Plant height [cm]**	**Root weight [g]**
Control_ FC	11.49^b^ ± 4.47	48.50^a^ ± 2.38	48.5^ab^ ± 6.25	97.00^ab^ ± 7.39	3.24^a^ ± 1.16
5% _FC	15.32^a^ ± 3.83	51.25^a^ ± 2.36	45.75^ab^ ± 8.22	97.00^ab^ ± 8.41	2.94^ab^ ± 0.66
10% _FC	11.25^b^ ± 3.42	50.25^a^ ± 2.06	36.25^b^ ± 6.95	86.50^b^ ± 7.55	2.96^ab^ ± 1.16
Control_NC	12.62^ab^ ± 2.97	47.75^a^ ± 3.86	39.25^b^ ± 6.85	87.00^b^ ± 9.63	2.95^ab^± 1.15
5% NC	8.53^b^ ± 1.69	49.25^a^ ± 0.96	37.50^b^ ± 8.74	86.75^b^ ± 8.96	1.04^b^ ± 0.48
10% NC	8.15^b^ ± 2.43	48.00^a^ ± 2.16	56.25^a^ ± 16.78	104.25^a^ ± 15.39	1.32^b^ ± 0.13

All the values are the mean of four replicate ± standard deviation. Values with different letters are statistically different (α = 0.05); **Control_FC** - seedlings inoculated with *F. culmorum* without extract, **5% _FC and 10% _FC** - seedlings treated with extracts and inoculated with *F. culmorum*, **Control_NC** - seedlings treated with water without the inoculation of *F. culmorum*, and **5% NC and 10% NC** - seedlings treated with extract without the inoculation of *F. culmorum*.

**Figure 4:**
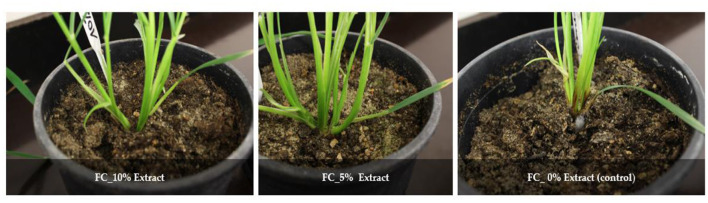
*F. culmorum* symptoms in the treated seedlings and the control group after 21 days of infection.

In summary, *L. album* flower extract and *F. culmorum* infection affected the whole plant. Five percent extract yielded 15.32 g of fresh weight of the seedling compared to 11.25 g for 10% extract. Furthermore, total plant height was also affected, and 5% of samples showed greater height than the 10% samples. In addition, visual differences between the seedlings were observed, with the infected seedlings displaying infection symptoms and non-infected wheat seedlings showing no symptoms (Figure 4).

### 2.3 *Fusarium* spp. identification

Molecular identification of all fungal strains that might be present in the collected seedlings was performed using sequence analysis of the PCR-amplified fragments PCR. The DNA regions were amplified with EF728M and Tef1α primers and were subsequently sequenced. Amplified DNA fragments were compared with reference genes from the GenBank Database to confirm species identification. The results showed that the isolated strains in the contaminated wheat seedlings were *Fusarium* spp., as expected. However, isolates in the roots of the control group were *F. culmorum* (Strains -FC1Croot, and FC-C2root with 96.4% and 96.81% identical bases, respectively). For roots of the seedlings treated with *L. album* extracts, the identified fungi were *F. oxysporum* (Strains - FC5.4.1R and FC10.5.1G with 97.86% and 98.2% identical bases, respectively). In addition, no significant similarities in fungal species were found in infected seedling leaves as well as in non-infected seedling leaves and roots (data not shown).

### 2.4 Effect of *L. album* flower extracts on *Fusarium* growth

The influence of extracts on the growth and development of *Fusarium* was measured by ergosterol (ERG) content, which is an indicator of fungal biomass. The HPLC/PDA analysis successfully detected the presence of ergosterol in both the roots and leaves of the harvested wheat seedlings (Table 3). ERG was present both in non-infected and infected seedlings. In the non-infected seedlings, the control without the extract showed the highest ERG level in leaves and roots, the ones with 5% of the extract being lower and the lowest for 10%, meaning that endophytic fungi were present in the plant tissue. In both cases (roots and leaves), the extract inhibited fungal development inside the plant. ERG levels in those seedlings were significantly lower compared to the infected seedlings. Similarly, in the case of artificially infected seedlings, the control without the extract had the highest level of ergosterol in roots and leaves. However by using the Equation 4, a significant ERG reduction was observed in the samples treated with 10% extract, with the highest reduction of up to 84.57% in the leaves and 65.52% in the roots. For the 5% extract, ergosterol was diminished by 52% in the roots and 79.31% in the leaves. This showed that the application of *L. album* extracts led to a notable lowering of ERG levels in the infected seedlings. Statistically, significant differences were observed between the 5% and 10% of extract, as determined by ANOVA with *post-hoc* Duncan test at *p* < 0.05.

**Table 3:** Comparative effects of *L. album* on the ergosterol content [μg/g] and its reduction [%] between the infected and non-infected wheat seedlings.

	**Ergosterol content [**μ**g/g]**		
**Treatments** ^*^	**Control**	**5% extract**	**10% extract**
Leaves_NI	9.89^d^ ± 1.62	8.81^cd^ ± 1.04 (10.94%)	7.72^cd^ ± 2.44 (21.92%)
Roots_NI	8.58^cd^ ± 0.39	5.51^cde^ ± 1.00 (35.80%)	4.85^de^ ± 0.80 (43.51%)
Leaves_I	25.11^b^ ± 4.17	5.19^cde^ ± 1.54 (79.31%)	3.87^e^ ± 1.83 (84.57%)
Roots_I	41.46^a^ ± 18.19	19.85^bc^ ± 7.34 (52.14%)	14.30^bcd^ ± 2.85 (65.52%)

All the values are the mean of four replicate ± standard deviation. Values with different letters are statistically different (α = 0.05); ^*^Leaves_NI: leaves of non-infected seedlings; **Roots_NI**: roots of non-infected seedling; **Leaves_I**: leaves of the infected seedlings; **Roots_I**: roots of the infected seedlings.

### 2.5 Effects of *L. album* flower extracts on mycotoxins biosynthesis in wheat seedlings artificially infected with *F. culmorum*

Mycotoxin biosynthesis is one of the challenging phenomena associated with fungal infection. Our study analyzed the effects of *L. album* flower extracts on the mycotoxins content in the roots and leaves of seedlings artificially infected with *F. culmorum* as well as in non-infected seedlings using the LC-MS/MS technique. Four commonly synthesized mycotoxins of *F. culmorum*, namely DON, 3- and 15-AcDON, ZEN, and ZEN-14S were quantified (Table 4). Notably, no mycotoxins were identified in the non-infected wheat seedlings.

**Table 4:** Effects of *L. album* on the mycotoxin biosynthesis [ng/g] on the infected wheat seedlings.

**Mycotoxins**	**Treatments**	**Seedling parts**	
		**Leaves_I**	**Roots_I**
DON	Control	nd^b^	18.90^a^ ± 13.37
	5%	nd^b^	nd^b^
	10%	nd^b^	nd^b^
3- and 15-AcDON	Control	nd^b^	30.31^a^ ± 12.10
	5%	nd^b^	nd^b^
	10%	nd^b^	nd^b^
ZEN	Control	31.30^b^ ± 6.70	56.01^a^ ± 8.48
	5%	nd^d^	10.00^c^ ± 2.48
	10%	nd^d^	7.28^c^ ± 1.73
ZEN-14S	Control	46.26^a^ ± 4.66	51.68^a^ ± 12.33
	5%	nd^c^	12.90^b^ ± 3.59
	10%	nd^c^	10.87^b^ ± 1.05

All the values are the mean of four replicates ± standard deviation. Values with different letters are statistically different (α = 0.05). ^*^Leaves_I: leaves of the infected seedlings; **Roots_I**: roots of the infected seedlings. DON, deoxynivalenol; 3- and 15-AcDON, 3- and 15-acetyl deoxynivalenol; ZEN, zearalenone; ZEN-14S, zearalenone-14-sulfate; nd, not detected.

A reduction of mycotoxin accumulation was noted in the seedlings treated with *L. album* flower extracts. In the roots, there were significantly higher amounts of all mycotoxins compared to the leaves, with lower production of DON and 3- and 15-AcDON than ZEN and ZEN-14S. *L. album* caused complete inhibition (100%) of DON and 3- and 15-AcDON in the roots. While ZEN was entirely inhibited in the leaves, in the roots decreased within the range of 10.00 to 7.28 ng/g compared to the control value of 56.01 ng/g, representing a reduction of 82.14% for the 5% and 87.41% for the 10% of *L. album* extract. Regarding ZEN-14S, complete inhibition occurred in the leaves, but in the roots, its concentration decreased within the range of 12.90 to 10.87 ng/g compared to the control value of 51.68 ng/g. This reduction was equivalent to 75.03% for 5% concentration and 78.97% for 10%. Overall, a significant impact of *L. album* on mycotoxin biosynthesis was shown, resulting in a noteworthy reduction in mycotoxin concentrations compared to the control, regardless of the extract concentration (differences were insignificant at *p* < 0.05 for all produced mycotoxins).

### 2.6 Correlation between ergosterol and the produced mycotoxins (Pearson r correlation)

**Figure 5:**
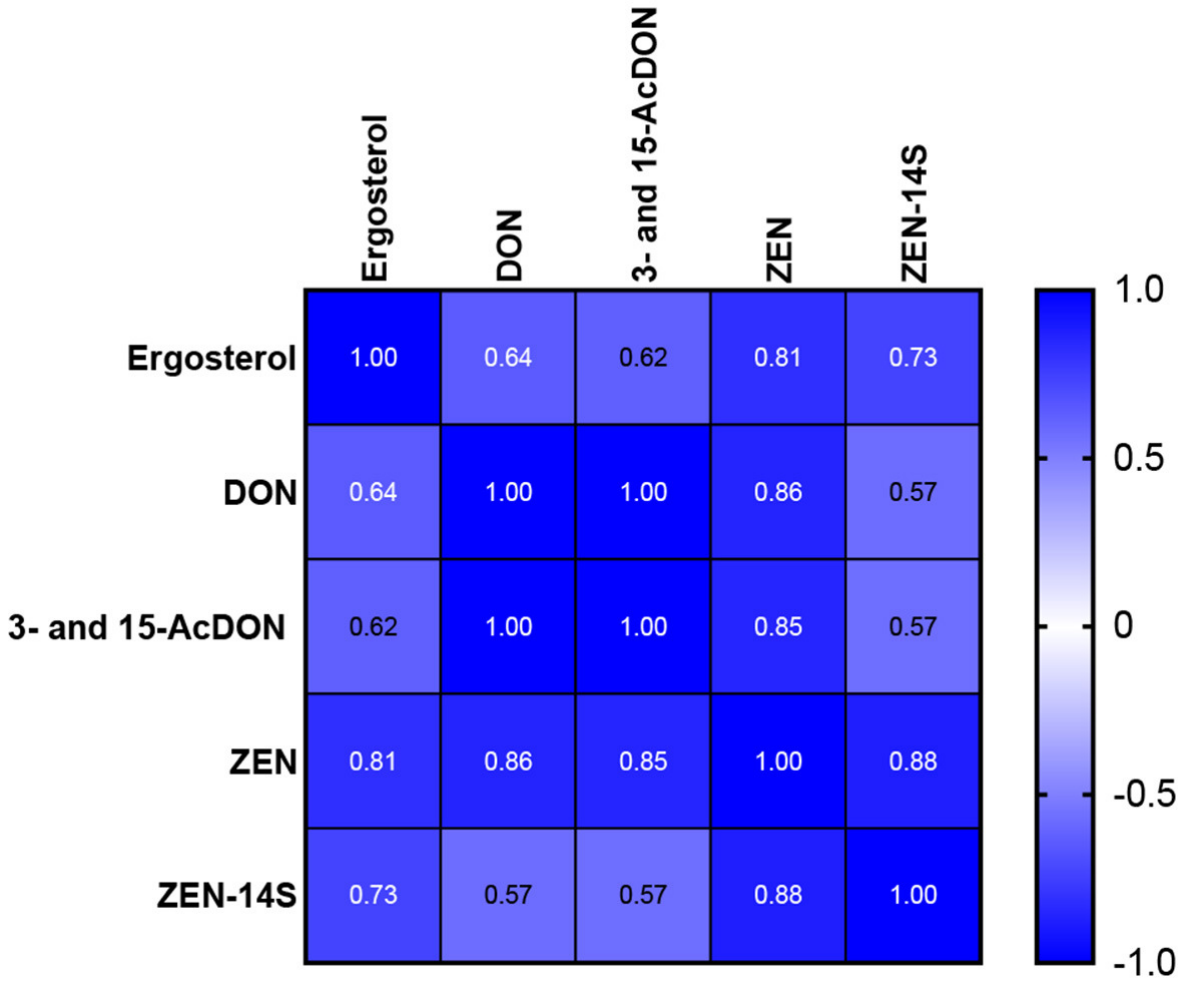
Pearson correlation between ergosterol content and biosynthesized mycotoxins in the infected wheat seedlings.

Pearson correlation (Pearson's r) assessed the linear relationship between ergosterol content and mycotoxins in the infected wheat seedlings. The analysis showed that ergosterol content positively correlated with the synthesized mycotoxins, and it was statistically significant at *p* < 0.05. Furthermore, highly positive correlations were found between the concentrations of DON and 3- and 15-AcDON and between ZEN and ZEN-14S (Figure 5). Compared to this, weaker positive correlations were observed between DON and ZEN-14S (*r* = 0.57).

## 3 Discussion

The germination capability of wheat seeds serves as a crucial indicator reflecting their quality, viability, and overall potential (Hassani et al., 2019). In our study, *L. album* decreased seed germination and seedling growth after seven days of incubation compared to the control. This effect was in agreement with other studies, showing reduced wheat germination of seeds treated with *Ulva linza* or *Corallina officinalis* seaweeds (Hamouda et al., 2022), *Flaveria bidentis* (Dai et al., 2022) and aqueous extracts of weed plants: *Hyptis sauveolens* (L.), *Ricinus communis* (L.)*, Alternanthera sessilis* (L.), *Ipomoea carnea* (Jacq), *Malachra capitata* (L.), and *Cymbopogon citrutus* (Stapf) (Joshi and Joshi, 2016). The decrease in germination percentage could be attributed to the potential phytotoxic or allelopathic effects of the extracts (Joshi and Joshi, 2016; Ma et al., 2011; Dai et al., 2022; Hamouda et al., 2022; Rys et al., 2022), such as the harmful or inhibitory impact that plant extracts may have on plant tissues or biological processes (Ma et al., 2011; Werrie et al., 2020) or chemical interactions between plants, which may be direct or indirect, beneficial or detrimental (Joshi and Joshi, 2016; Aurelio et al., 2022). The control group exhibited the longest wheat seedlings, with the lowest recorded dry weight compared to the treated wheat seeds. Although plant extracts mildly inhibited seedling weight loss (Figure 1), the effect was not significantly different from the control at *p* < 0.05. The observed reduction in plant growth under natural conditions may be attributed to seed competition for water, minerals, oxygen, or root system space (Rys et al., 2022). Some plant extracts, despite their beneficial properties, can also contain compounds that hinder seed germination or seedling growth (Bota et al., 2021). Hamouda et al. (2022) investigated the effect of wheat seed priming with the aqueous extracts of *Ulva linza* and *Corallina officinalis* seaweeds; higher concentrations inhibited seedling growth and caused specific chromosomal abnormalities, while low concentrations of seaweed extract as a priming treatment improved wheat seedling growth, physiological features and had a greater capacity to boost bioavailable macro- and micronutrients to the plant (Hamouda et al., 2022).

*F. culmorum* can impact plants across different developmental stages, leading to seedling blight and root rot, hindering seedling emergence and overall plant development (Pastuszak et al., 2021). This pathogenic fungus infects wheat plants, leading to foot rot symptoms, particularly affecting the roots and lower stems. The severity of the disease varies, with early infections causing pre- and post-emergence seedling death, while later infections result in brown lesions on the lower stems and tiller abortion (Scherm et al., 2013). While FHB is often a primary focus in research due to its economic impact and mycotoxin production (Bottalico and Perrone, 2002; Scherm et al., 2013; Antalová et al., 2020), foot rot caused by *F. culmorum* is a significant issue that requires attention (Scherm et al., 2013). The presence of *L. album* and *F. culmorum* infection caused stress to the growing seedlings and by responding to these biotic stresses, seedlings produced many short roots that have influenced the weight and length of the roots (Table 2). Roots are crucial for nutrient uptake and overall plant health. The observed decrease in root length for the seedling treated with 10%_FC may be a defense response triggered by the plant in the presence of *Fusarium*. *L. album* extract might influence this response positively, aiding the wheat seedling in resisting fungi infection. Moreover, shorter roots may limit the pathogen's ability to invade the plant's vascular system, acting as a physical barrier. While a decrease in root length might be perceived as an adverse effect of *Fusarium* infection in wheat, as noted in a study conducted by Saad et al. (2023), it could be a strategic adaptation to strengthen the plants' defense mechanisms induced by plant extract against *Fusarium* spp, ultimately contributing to enhanced resistance. Previous research showed that seedlings exhibiting moderate vigor were more susceptible to infection than those with high vigor and exhibited a significant height increase when subjected to inoculation with *F. graminearum* spores (Zhou et al., 2018). Similarly, the application of essential oil disturbed the growth of seedlings' roots and reduced the vigor index (Grzanka et al., 2021). Seedlings protected with *L. album* extracts showed no symptoms. In contrast, the control samples exhibited visible symptoms, such as root browning, yellowing of the first leaves, and visible mycelia on the above-ground part of the seedlings (Figure 4). Previous studies reported that the early infection reduced wheat seedling growth as *F. culmorum* can effectively penetrate seedling roots, migrate from hypocotyl to the upper stem internodes and leaves, colonize the host's tissue and cells, block the vascular bundles, disturb nutritional supply and metabolic processes (Kthiri et al., 2021). In another study, three durum wheat genotypes at seedling and full anthesis stage *F. culmorum* infection significantly decreased chlorophyll a, b, and carotenoid contents in the leaves (Pastuszak et al., 2021). *L. album* may also cause adverse effects against *F. culmorum*, inducing toxin accumulation, especially under conditions unfavorable for fungal growth or at low extract concentrations (Tretiakova et al., 2022).

Ergosterol, being integral to fungal cell membranes, serves as an indicator of fungal biomass and is vital for maintaining cell function and integrity (Perkowski et al., 2008; Uwineza et al., 2022). Severe ERG depletion may influence cell growth and proliferation (Abhishek et al., 2015). Here, the effect of *L. album* extract was evident, as shown by the ERG reduction in infected seedlings. Low ERG levels in non-infected seedlings could be attributed to endophytic fungi—microorganisms living within plant tissues without visible disease symptoms (Abaya et al., 2021; Noel et al., 2022). PCR-based identification confirmed *F. oxysporum* in the root of infected samples treated with *L. album* flower extracts. Also, no mycotoxins were identified in those samples. Noel et al. (2022) reported *F. oxysporum* as one of the fungal endophytes that significantly increased seed weight and lowered the accumulation of DON compared to *F. graminearum*-infected wheat heads without endophyte (Noel et al., 2022). *L. album* extract suppressed the growth of pathogenic and endophytic fungi in a concentration-dependent manner. This inhibitory effect could be attributed to the active compounds in *L. album* flower extracts (Pereira et al., 2012; Pourmirzaee et al., 2019; Sulborska et al., 2020; Uwineza et al., 2021). Detrimental impact on ERG content confirmed a significant disruption or biosynthesis blockage within the plasma membrane of *F. culmorum*. This finding is in agreement with some of the previous studies that have reported a decrease in ERG content in infected samples after the application of plant extracts (Abhishek et al., 2015; Uwineza et al., 2022). Leaf extract of *Solanum torvum* Swartz. decreased ERG production by the *Fusarium verticillioides* strain (Abhishek et al., 2015). Previous *in vitro* study also showed *L. album* flower extract activity against *F. culmorum* and *F. proliferatum*, reducing ERG content (Uwineza et al., 2023).

Mycotoxigenic fungi can simultaneously produce various compounds (Streit et al., 2012). To monitor whether *L. album* extract alters the mycotoxin biosynthesis in wheat seedlings, an analysis was conducted, revealing that the content of mycotoxins, including DON, 3- and 15-AcDON, ZEN, and ZEN-14S, commonly associated with *F. culmorum* contamination (Bryła et al., 2018; Uwineza et al., 2022), were reduced both in roots and leaves of seedlings treated with *L. album* extract. Abbas and Yli-mattila (2022) showed that the methanolic extract of the medicinal plant *Zanthoxylum bungeanum* successfully decreased *F. graminearum* growth and abrogated DON production in wheat heads (Abbas and Yli-mattila, 2022). Similarly, Shcherbakova et al. (2018) found no increase in DON, AcDON, and ZEN content when *F. culmorum* and *F. graminearum* developed on extract-treated wheat seeds and seedlings (Shcherbakova et al., 2018).

DON and its acetylated forms 3- and 15-AcDON were completely inhibited by *L. album*. Correlations between DON and its acetylated forms were highly significant, which is in accordance with the previous research (Sunic et al., 2021), where DON highly correlated with 3-AcDON in winter wheat, and its production by *F. culmorum* is believed to play a role in pathogenesis (Morimura et al., 2020). Mycotoxin accumulation affects germination rates, seedling growth, pathogen aggressiveness, and overall disease severity (Bruins et al., 1993; Scherm et al., 2011; Winter et al., 2019). Scherm et al. (2011) showed that DON is an aggressiveness factor in *F. culmorum* stem base infections of durum wheat, whereby disease incidence was decreased by 40%−80% when the trichothecene regulatory gene *Tri6* was silenced (Scherm et al., 2011). Similarly, Winter et al. (2019) showed that high DON- and 3-AcDON-producing isolate led to more severe symptoms and 20 times more significant colonization of the stem base than isolates that produced less DON/3-AcDON (Winter et al., 2019). A 10-fold higher accumulation of ZEN and ZEN-14S in infected seedling roots was observed compared to DON, which is consistent with previous findings (Ksieniewicz-Wozniak et al., 2021). It can be explained by *L. album* extract ability to regulate the enzymes involved in the synthesis or modification of ZEN and its derivatives compared to the ones that process DON. Additionally, the accumulation of ZEN and ZEN-14S over DON may be favored, according to a study that investigated the effect of compactin on mycotoxin production and the expression of associated biosynthetic and regulatory genes in toxigenic *F. culmorum*. Their findings showed that compactin had a suppressing effect on DON and ZEN differently due to its effect on various genes involved in the biosynthesis of these mycotoxins (Stakheev et al., 2022). These intricate interactions could lead to differential expression of genes related to mycotoxin biosynthesis and modification, resulting in the observed variations in mycotoxin levels (Scherm et al., 2011). However, even though ZEN and ZEN-14S were highly synthesized, the application of *L. album* significantly reduced them compared to the control. ZEN was reduced to 82.14% for 5% and 87.41% for 10% of *L. album* extract, and ZEN-14S was decreased to 75.03% for 5% and 78.97% for 10%, confirming the mechanism of action of *L. album* extract on the reduction of mycotoxin biosynthesis (Jafarzadeh et al., 2022). A strong correlation between ZEN and ZEN-14S was observed, while a weaker correlation occurred among DON, ZEN, and ZEN-14S, which is in accordance with previous research (Birr et al., 2021). ZEN can be modified (e.g., via conjugation) in plants, fungi, and animals through phase I and phase II metabolism (Veršilovskis et al., 2019). This conjugated form (ZEN-14S) is approximately 60 times more estrogenic than ZEN and can be easily hydrolyzed to ZEN in the gastrointestinal tract, thereby increasing exposure to ZEN (Veršilovskis et al., 2019). A high ZEN-14S/ZEN ratio in the malted wheat suggests a possibility of *Fusarium* converting ZEN into a phase II metabolite through sulfation reactions (Ksieniewicz-Wozniak et al., 2021). Wheat infections occur during or shortly after flowering (Wegulo, 2012); however, our study revealed that mycotoxin accumulation can start at the initial stages of infection.

In this study, we have presented the dual effect of *L. album* extracts, which acted as antifungal on *F. culmorum* in wheat seedlings and showed the phytotoxic effect on seed germination. Composition and concentration of *L. album* extracts can be essential here, as they have not been analyzed until now, but similar results have been reported previously (Bayar and Yilar, 2019; El-Alam et al., 2020; Mehdizadeh et al., 2020). El-Alam et al. (2020) reported that all tested essential oils (EO) presented antifungal properties against *F. culmorum* by direct contact and volatility assays. However, all tested EO have also shown a phytotoxic activity, either by inhibiting seed germination or affecting root elongation of rye and lettuce (El-Alam et al., 2020). Therefore, carefully considering and optimizing the extract concentration and application method is advised to ensure that the antifungal benefits outweigh any potential phytotoxic effects of the plant extracts.

## 4 Conclusions

The present study highlights the dual impact of *L. album* flower extracts on wheat seed germination, seedling growth, and *Fusarium* infection control. While exhibiting phytotoxic and allelopathic effects that hindered seed germination and seedling growth, the extracts effectively controlled *F. culmorum* infection and reduced visible symptoms. The anti-mycotoxigenic potential of *L. album* flower extracts was evident in the complete inhibition of DON, 3- and 15-AcDON, and the substantial reduction of ZEN and ZEN-14S in artificially infected wheat seedlings. The reduction of ergosterol content in the seedling roots confirmed the disruptive impact on *F. culmorum*, emphasizing the potential of *L. album* as a biocontrol agent against *Fusarium* mycotoxins. The study highlights intricate molecular mechanisms at play and emphasizes the need for further exploration of the effect of *L. album* extracts on key regulatory genes for mycotoxin biosynthesis. Despite the observed phytotoxicity, the study suggests the potential of *L. album* extracts as a biocontrol agent, stressing the importance of optimizing concentration and application methods in agricultural practices. Overall, this research contributes valuable insights into the sustainable use of *L. album* in combating *Fusarium* species in wheat cultivation, with further exploration required for effective implementation.

## 5 Materials and methods

### 5.1 Plant extraction

The material used for the extraction was dried *Lamium album* flowers purchased from a certified Polish company called Dary Natury located in Podlaskie Voivodeship of Poland (53°4′10.98 latitude and 22°58′2.87 longitude). The extraction method used was the supercritical fluid extraction technique using methanol as a co-solvent, as described in the previous study (Uwineza et al., 2022). Subsequently, the obtained extracts were evaporated in a vacuum evaporator (Buchi R-215 Rotary Evaporator System, Germany) at 40°C to eliminate the methanol; the dried extract was reconstituted in a measured volume of distilled water and stored at −18°C until antifungal assays.

### 5.2 Studied material

Healthy wheat grains (*Triticum aestivum* L. cultivar Arkadia) were provided by the Plant-Pathogen Interaction Team, Institute of Plant Genetics, Polish Academy of Sciences, Poznan, Poland. Fungal isolate of *F. culmorum* KF 846 was obtained from the collection of the Plant-Pathogen Interaction Team, Institute of Plant Genetics, Polish Academy of Sciences, Poznan, Poland. The tested strain was cultured in 9 cm Petri dishes on potato dextrose agar medium (PDA, BioShop, Burlington, ON, Canada) at 28°C for 7 days.

### 5.3 Standards, chemicals and reagents

Carbon dioxide (CO_2_, SFE grade), contained in a dip tube cylinder, was purchased from Air Products Sp, Poland. Methanol for HPLC-super gradient was purchased from POCh (Gliwice, Poland). Acetonitrile, methanol, and water for LC-MS grade were acquired from POCh (Gliwice, Poland). Dream Taq green PCR master mix kit was purchased from Thermo Scientific (Vilnius, Lithuania). Analytical standards including ERG, ZEN, DON, 15-AcDON, and 3-AcDON were purchased in ready-to-use solutions from Romer Labs (Tulln, Austria), and ZEN-14S (100 μg/mL) purchased in Aokin (Berlin, Germany). Depending on solubility, the standards were dissolved in acetonitrile. All standards were stored in amber glass vials at approximately −20°C. A mixture of all standards necessary for a particular analytical run was prepared immediately before the analysis.

### 5.4 Effect of *Lamium album* flower extract on seed germination and seedling growth

The effect of *Lamium album* flower extract on the germination of wheat kernels was determined according to Al-Khafajy et al. (2022) with some modifications. Ten mL solution of *L. album* flower extract was prepared at 5% and 10% concentrations, then added to sterilized glass plates with tissue paper containing sterile 10 grains/plate; the control samples were prepared with 10 mL of distilled water, and aseptically prepared plates (four plates for each treatment) were incubated under controlled whirlpool chamber at 28°C for 7 days. Germination percentage was calculated using the following formula (Equation 1):


(1) $$\begin{array}{c} \text{Germination percentage (\%)}=\\ \text{(number of healthy seedlings/total number of the seed)}\times 100\; \end{array}$$

The root and shoot length of the seedlings were measured after seven days of germination. Then, all seedlings were collected and freeze-dried to determine the seedling dry weight (g). Also, seedling length vigor index (SLVI) and seedling weight vigor index (SWVI) relations were determined by the following Equations 2, 3 (Hassani et al., 2019):


(2) $$\begin{array}{c} \text{SLVI}=\left(\text{mean shoot length (cm)}+\text{mean root length (cm)}\right)\\ \times \text{percentage of seed germination)} \end{array}$$

(3) $$\begin{array}{l} \text{SWVI}=\text{dry weight seedling (g)}\times \text{percentage of seed germination} \end{array}$$

### 5.5 Effect of *Lamium album* flower extracts on wheat seedlings against *F. culmorum* in controlled conditions

Ten wheat grains were surface sterilized using a 0.6% (v/v) bleach (sodium hypochlorite) solution, followed by rinsing with distilled water three to four times. Subsequently, 10 mL of *Lamium album* flower extract was prepared at 5% and 10% concentrations, then added to sterilized glass plates with tissue paper containing 10 sterile grains/plate. The control samples were prepared with 10 mL of distilled water. Aseptically prepared plates (four plates for each treatment) were incubated under a controlled whirlpool chamber at 28°C for 7 days to germinate. Plastic pots (13 cm in height) were prepared and filled with a sterile potting mix of soil, sand, and horticulture vermiculite fine (60%, 30%, and 10%; v/v/v) each. Each pot was then planted with a germinated grain (7-day-old seedling) treated with water, and grains treated with *L. album* extracts at 5 and 10% were marked accordingly. The pots were divided into two groups (four pots for each treatment): the first group served as the control, while in the second group, each pot was inoculated with 100 ml of the prepared spore suspension of *F. culmorum* containing 10^6^ conidial spores/mL after 10 days of potting. All the pots were arranged on the control room benches, maintained under a natural photoperiod (16 h of light/8 h of darkness) at 23 ± 4°C and with 45% humidity. The seedlings were irrigated with sterile water (100 ml) after 2 days. Daily observations were made to detect any disease symptoms, and the experiment was concluded on the 21^st^ day after inoculation. Both underground (roots) and aerial parts (leaves) of the seedlings were measured (length and weight), collected separately, and stored for further analysis. The experiment comprised four replicates for each treatment, including infected and non-infected wheat seedlings treated with *L. album* flower extracts at 0% control, as well as 5% and 10% concentrations. This process was repeated for reproducibility, and the results represent the mean of two distinct experiments.

#### 5.5.1 DNA extraction, molecular identification, PCR primers, and DNA sequencing

##### 5.5.1.1 Isolation and purification of fusarium species

To isolate *Fusarium* spp. from the collected seedling parts (roots and leaves) from each treatment, a 0.6% (v/v) bleach (sodium hypochlorite) solution was used for surface sterilization, followed by rinsing with distilled water three to four times. Subsequently, the sterilized samples were placed on sterile plates with PDA and incubated at 28°C in the dark for 7 days. The resulting colonies were transferred until purified isolates were obtained. All fungi were maintained on PDA medium at 4°C and sub-cultured monthly until DNA analysis.

##### 5.5.1.2 DNA extraction and *Fusarium* sp. identification

Genomic DNA was extracted using a modified method with the hexadecyltrimethylammonium bromide (CTAB) (Urbaniak et al., 2020). Mycelium scraped from a 7-day-old PDA culture was ground to a fine powder with liquid nitrogen, and 800 μl of CTAB solution was added to each Eppendorf tube, 150 μl of chloroform-isoamyl alcohol (24:1), then mixed gently. Then, 4 μl of β-mercaptoethanol was added, and samples were incubated in the water bath for 20 min at 65°C. After cooling for 5 min, 150 μl of chloroform-isoamyl alcohol (24:1) was added and mixed gently. The samples were centrifuged for 20 min at 12,000 rpm and 4°C. The supernatant was supplied with 60 μl of 3M sodium acetate and 1,000 μl of ethyl alcohol and precipitated 20 min at −23°C. Then, the samples were centrifuged for 20 min at 12,000 rpm, air dried for 1 h, and re-dissolved in 150 μl of TE buffer pH 8.

Polymerase chain reactions (PCRs) were carried out using Dream Taq green PCR master mix with the help of a C-1000 Touch thermal cycler (Bio-Rad, Hercules, CA, USA). The conditions for PCR amplification were described earlier (Urbaniak et al., 2020), where primers EF728M (5′-CATCGAGAAGTTCGAGAAGG-3′) and Tef1α (5′-GCCATCCTTGGAGATACCAGC-3′) were used. Each reaction tube contained 25 μl reaction mixture made of 12.5 μl of Dream Taq green PCR master mix (Thermo Scientific, Vilnius, Lithuania), 11 μl of water, nuclease-free (Thermo Fisher Scientific, Vilnius, Lithuania), 0.1μl of each primer pairs (EF728M and Tef1α; Thermo Fisher Scientific, Vilnius, Lithuania), and 1.5μl of DNA extract samples. The amplification conditions were as follows: initial denaturation of 3 min at 95°C, 35 cycles of (30 s at 95°C, 30 s at 56°C), and 1 min at 72°C with a final elongation of 15 min at 72°C. Amplification products were electrophoresed in 1.5% agarose gels (EURx Ltd., Gdansk, Poland) in 1 × TBE buffer (0.178 mol L-1 Tris-borate, 0.178 mol L-1 boric acid, 0.004 mol L-1 EDTA from Sigma-Aldrich, Steinheim, Germany) containing 5 μl of ethidium bromide. Fragments were visualized under a UV transilluminator and photographed using a PolyDoc System. GeneRuler TM 100bp DNA Ladder Plus was used to establish the molecular weight of the products.

For sequence analysis, PCR-amplified DNA fragments were purified with exonuclease I (Vilnius, Lithuania) and Fast AP thermosensitive alkaline phosphatase (Vilnius, Lithuania) using the following program: 15 min at 37°C and 15 min at 85°C. The strand was labeled using a BigDye^Tm^ Terminator v3.1 Cycle Sequencing Kit (Thermo Fisher Scientific, Vilnius, Lithuania), according to the previously described protocol (Stepień and Waśkiewicz, 2013) and precipitated with 70% ethanol. Sequence reading was performed using Applied Biosystems equipment. Sequences were edited using Chromas v. 1.43 (Technelysium, Tewantin, Australia) and analyzed using the BLASTn algorithm. Sequences were deposited in GenBank and will be publicly available.

#### 5.5.2 Chemical analysis

##### 5.5.2.1 The ERG content analysis

After collecting the seedling's roots and leaves, the materials were lyophilized and ground into fine powder. The concentration of ERG in the samples was determined by comparing the retention time of the analyte with that of an external standard (Waśkiewicz et al., 2014). The method had a detection limit of 10 ng/g. The ergosterol reduction percentage was calculated using the following formula (Equation 4):


(4) ERGreduction(%)=[(control-treatment)/control]×100

##### 5.5.2.2 Mycotoxins analysis

Mycotoxin extraction was performed by adding 5 mL of the extraction solvents (acetonitrile: water, 86:16, v/v) to 0.5 g of dried roots or leaves of the infected and non-infected wheat seedlings, vortexing (for about 30 s) and mixing using a horizontal shaker for 24 h. After extraction, the samples were centrifuged at 7,500 rpm for 10 min. Then, approximately 2 mL of supernatant was filtered through a 0.2 μm syringe filter (Chromafil, Macherey-Nagel, Duren, Germany) and poured into vials for chromatographic analysis. For the analysis, the method reported by Uwineza et al. (2022) was followed with some modifications (Perczak et al., 2020). The compounds were quantitatively analyzed using multiple reaction monitoring. The mycotoxin concentrations (μg/g) were calculated using a calibration curve based on commercial single-component preparations of DON, 3- and 15-AcDON, ZEN, and ZEN-14S. All samples were analyzed in triplicate.

#### 5.5.3 Statistical analysis

The experimental design consisted of four replicates of each treatment, and each experiment was repeated twice. The results were interpreted as mean standard deviation. Analysis of variance (ANOVA-One way) was applied for the statistical analysis of experimental data using the Statgraphics v. 4.1 software package (Graphics Software System, STCC, Inc., Rockville, MD, USA), and each experimental value was compared with the corresponding control. Where there was statistical significance (*p* < 0.05), the mean values were further separated using Duncan's multiple-range test. GraphPad Prism9 was used for the graphs.

## Data availability statement

The raw data supporting the conclusions of this article will be made available by the authors, without undue reservation.

## Author contributions

PAU: Conceptualization, Data curation, Formal analysis, Investigation, Methodology, Project administration, Software, Validation, Visualization, Writing – original draft, Writing – review & editing. MU: Formal analysis, Investigation, Methodology, Validation, Visualization, Writing – original draft. ŁS: Resources, Writing – review & editing. AG-M: Data curation, Validation, Writing – review & editing. AW: Conceptualization, Data curation, Methodology, Resources, Supervision, Writing – original draft, Writing – review & editing.

## References

[B1] Abaya A. Serajazari M. Hsiang T. (2021). Control of *Fusarium* head blight using the endophytic fungus, *Simplicillium Lamellicola*, and its effect on the growth of *Triticum Aestivum*. Biol. Control 160:104684. 10.1016/j.biocontrol.2021.104684

[B2] Abbas A. Yli-mattila T. (2022). Biocontrol of *Fusarium Graminearum*, a causal agent of *Fusarium* head blight of wheat, and deoxynivalenol accumulation: from *in vitro* to *in planta*. Toxins (Basel) 14:299. 10.3390/toxins14050299 35622546 PMC9143666

[B3] Abdallah M. F. Ameye M. Saeger S. De; Audenaert K. Haesaert G. (2018). Biological Control of mycotoxigenic fungi and their toxins: an update for the pre-harvest approach,” in *Mycotoxins-Impact and Management Strategies*, eds. Njobeh P. B. Stepman F. (London: IntechOpen). 10.5772/intechopen.76342

[B4] Abhishek R. U. Thippeswamy S. Manjunath K. Mohana D. C. (2015). Antifungal and antimycotoxigenic potency of solanum torvum swartz. Leaf extract: isolation and identification of compound active against mycotoxigenic strains of aspergillus flavus and fusarium verticillioides. J. Appl. Microbiol. 119, 1624–1636. 10.1111/jam.12956 26394117

[B5] Acheuk F. Basiouni S. Shehata A. A. Dick K. Hajri H. Lasram S.. (2022). Status and prospects of botanical biopesticides in europe and mediterranean countries. Biomolecules 12, 1–30. 10.3390/biom12020311 35204810 PMC8869379

[B6] Alisaac E. Mahlein A.-K. Alisaac E. Mahlein A.-K. (2023). *Fusarium* head blight on wheat: biology, modern detection and diagnosis and integrated disease management. Toxins 15:192. 10.3390/toxins15030192 36977083 PMC10053988

[B7] Al-Khafajy M. J. Majeed H. A. Mutlag N. A. Cheyed S. H. (2022). Wheat seed deterioration stimulated by plant extracts. Bionatura 7, 1–4. 10.21931/RB/2022.07.04.15

[B8] Antalová Z. Bleš D. Martinek P. Matuš Insky Id P. (2020). Transcriptional analysis of wheat seedlings inoculated with *Fusarium culmorum* under continual exposure to disease defence inductors. PLoS ONE 15:e0224413. 10.1371/journal.pone.0224413 32045412 PMC7012390

[B9] Aurelio S. Mauromicale G. Caruso P. Lombardo S. L. (2022). Allelopathy in durum wheat landraces as affected by genotype and plant part. Plants 8:1021. 10.3390/plants11081021 35448748 PMC9026900

[B10] Bayar Y. Yilar M. (2019). The antifungal and phytotoxic effects of different extracts of Salvia Virgata jacq. F. Env. Bullet. 4, 3492–3497.

[B11] Birr T. Jensen T. Preußke N. Sönnichsen F. D. Boevre M. De; Saeger S.. (2021). Occurrence of *Fusarium* mycotoxins and their modified forms in forage maize cultivars. Toxins (Basel) 13:110. 10.3390/toxins13020110 33540691 PMC7913079

[B12] Bota V. Sumalan R. M. Raba D. N. Negrea M. Poiana M.-A. Obistioiu D.. (2021). Fungi and mycotoxins control of wheat grains using essential oils. Sci. Pap. D-Animal Sci. 64, 325–329.

[B13] Bottalico A. Perrone G. (2002). Toxigenic *Fusarium* species and mycotoxins associated with head blight in small-grain cereals in Europe. Eur. J. Plant Pathol. 108, 611–624 10.1007/978-94-010-0001-7_2

[B14] Bruins M. B. M. Karsaï I. Schepers J. Snijders C. H. A. (1993). Phytotoxicity of deoxynivalenol to wheat tissue with regard to *in Vitro* Selection for *Fusarium* head blight resistance. Plant Sci. 94, 195–206. 10.1016/0168-9452(93)90020-Z

[B15] Bryła M. Waskiewicz A. Ksieniewicz-Wozniak E. Szymczyk K. Edrzejczak R. J. (2018). Modified *Fusarium* mycotoxins in cereals and their products—metabolism, occurrence, and toxicity: an updated review. Molecules 23, 1–34. 10.3390/molecules23040963 PMC6017960 29677133

[B16] Castro J. C. Pante G. C. Centenaro B. M. Almeida R. T. R. De Pilau E. J. Dias Filho B. P.. (2020). Antifungal and antimycotoxigenic effects of *Zingiber Officinale, Cinnamomum Zeylanicum and Cymbopogon Martinii* essential oils against *Fusarium Verticillioides*. Food Addit. Contamin. 37, 1531–1541. 10.1080/19440049.2020.1778183 32684097

[B17] Chipeva A. V; Geneva M. E. Dimitrova M. A. Penka; Moncheva A. Kapchina-Toteva M. V. (2013). Antimicrobial activity of extracts from *in vivo* and *in vitro* propagated *Lamium album* l. Plants. Afr. J. Tradit. Compl. Altern. Med. 10, 559–562. 10.4314/ajtcam.v10i6.30 24311888 PMC3847403

[B18] Dai L. Wu L. Zhou X. Jian Z. Meng L. Xu G. (2022). Effects of water extracts of *Flaveria bidentis* on the seed germination and seedling growth of three plants. Sci. Rep. 12, 1–7. 10.1038/s41598-022-22527-z 36271248 PMC9587036

[B19] Damtoft S. (1992). Iridoid glucosides from *Lamium album*. Phytochemistry 31, 175–178. 10.1016/0031-9422(91)83030-O

[B20] Deresa E. M. Diriba T. F. (2023). Phytochemicals as alternative fungicides for controlling plant diseases: a comprehensive review of their efficacy, commercial representatives, advantages, challenges for adoption, and possible solutions. Heliyon 9:e13810. 10.1016/j.heliyon.2023.e13810 36879959 PMC9984788

[B21] El-Alam I. Raveau R. Fontaine J. Verdin A. Laruelle F. Fourmentin S.. (2020). Antifungal and phytotoxic activities of essential oils: *in vitro* assays and their potential use in crop protection. Agronomy 10, 1–19. 10.3390/agronomy10060825

[B22] García-Ramírez E. Contreras-Oliva A. Salinas-Ruiz J. Hernández-Ramírez G. Spinoso-Castillo J. L. Cuevas S. I. C. (2023). Plant extracts control *in vitro* growth of disease-causing fungi in chayote. Plants 12:1800. 10.3390/plants12091800 37176858 PMC10180525

[B23] Gebremariam E. S. Karakaya A. Erginbas-Orakci G. Dababat A. A. Paulitz T. C. (2020). Assessment of the seedling resistance of spring wheat lines to *Fusarium Culmorum*. Tarim Bilim. Derg. 26, 87–93. 10.15832/ankutbd.466442

[B24] Grzanka M. Sobiech Ł. Danielewicz J. Horoszkiewicz-Janka J. Skrzypczak G. Sawinska Z.. (2021). Impact of essential oils on the development of pathogens of the *fusarium* genus and germination parameters of selected crops. Open Chem. 19, 884–893. 10.1515/chem-2021-0079

[B25] Hamouda M. M. Saad-Allah K. M. Gad D. (2022). Potential of seaweed extract on growth, physiological, cytological, and biochemical parameters of wheat *(Triticum Aestivum L.)* seedlings. J. Soil Sci. Plant Nutr. 22, 1818–1831. 10.1007/s42729-022-00774-3

[B26] Hassani F. Zare L. Khaledi N. (2019). Evaluation of germination and vigor indices associated with *Fusarium*-infected seeds in pre-basic seeds wheat fields. J. Plant Prot. 59, 69–85. 10.24425/jppr.2019.126037

[B27] Iqbal M. J. Shams N. Fatima K. Iqbal M. J. Shams N. Fatima K. (2022). “Nutritional quality of wheat,” in Wheat- Recent Advances (London: IntechOpen).

[B28] Jafarzadeh S. Abdolmaleki K. Javanmardi F. Hadidi M. Mousavi Khaneghah A. (2022). Recent advances in plant-based compounds for mitigation of mycotoxin contamination in food products: current status, challenges and perspectives. Int. J. Food Sci. Technol. 57, 2159–2170. 10.1111/ijfs.15555

[B29] Joshi N. Joshi A. (2016). Allelopathic effects of weed extracts on germination of wheat. Ann. Plant Sci 5:1330. 10.21746/aps.2016.05.001

[B30] Ksieniewicz-Wozniak E. Bryła M. Michałowska D. Waśkiewicz A. Yoshinari T. (2021). Transformation of selected *Fusarium* toxins and their masked forms during malting of various cultivars of wheat. Toxins (Basel) 13:866. 10.3390/toxins13120866 34941704 PMC8707366

[B31] Kthiri Z. Jabeur M. Ben; Chairi F. López-Cristoffanini C. López-Carbonell M. Serret M. D.. (2021). Exploring the potential of *Meyerozyma Guilliermondii* on physiological performances and defense response against *Fusarium* crown rot on durum wheat. Pathogens 10, 1–14. 10.3390/pathogens10010052 33429997 PMC7827111

[B32] Kursa W. Jamiołkowska A. Wyrostek J. Kowalski R. (2022). Antifungal effect of plant extracts on the growth of the cereal pathogen *Fusarium* spp.—an *in vitro* study. Agronomy 12:3204. 10.3390/agronomy12123204

[B33] Ma L. Wu H. Bai R. Zhou L. Yuan X. Hou D. (2011). Phytotoxic effects of *Stellera Chamaejasme* L. root extract. African J. Agric. Res. 6, 1170–1176. 10.5897/AJAR10.732

[B34] Mehdizadeh L. Taheri P. Ghasemi Pirbalouti A. Moghaddam M. (2020). Phytotoxicity and Antifungal Properties of the Essential Oil from *the Juniperus Polycarpos Var. Turcomanica (B. Fedsch.) R. P. Adams Leaves*. Physiol. Mol. Biol. Plants 26, 759–771. 10.1007/s12298-020-00776-4 32255938 PMC7113358

[B35] Morimura H. Ito M. Yoshida S. Koitabashi M. (2020). Toxins *in vitro* assessment of biocontrol effects on *Fusarium* head blight and deoxynivalenol (DON) accumulation by DON-degrading bacteria. Toxins (Basel) 12, 1–13. 10.3390/toxins12060399 32560237 PMC7354482

[B36] Noel Z. A. Roze L. V. Breunig M. Trail F. (2022). Endophytic fungi as a promising biocontrol agent to protect wheat from *Fusarium Graminearum* head blight. Plant Dis. 106, 595–602. 10.1094/PDIS-06-21-1253-RE 34587775

[B37] Ostry V. Malir F. Toman J. Grosse Y. (2017). Mycotoxins as human carcinogens—the IARC monographs classification. Mycotoxin Res. 33, 65–73. 10.1007/s12550-016-0265-7 27888487

[B38] Özdemir F. (2022). Host susceptibility of CIMMYT's international spring wheat lines to crown and root rot caused by *Fusarium Culmorum* and *F. Pseudograminearum*. Agronomy 12:3038. 10.3390/agronomy12123038

[B39] Pastuszak J. Szczerba A. Dziurka M. Hornyák M. Kopeć P. Szklarczyk M.. (2021). Physiological and biochemical response to *Fusarium Culmorum* infection in three durum wheat genotypes at seedling and full anthesis stage. Int. J. Mol. Sci. 22:7433. 10.3390/ijms22147433 34299055 PMC8303160

[B40] Perczak A. Gwiazdowska D. Gwiazdowski R. Juś K. Marchwińska K. Waśkiewicz A. (2020). The inhibitory potential of selected essential oils on *Fusarium* spp. Growth and mycotoxins biosynthesis in maize seeds. Pathogens 9:23. 10.3390/pathogens9010023 31887989 PMC7168669

[B41] Pereira O. R. Domingues M. R. M. Silva A. M. S. Cardoso S. M. (2012). Phenolic constituents of lamium album : focus on isoscutellarein derivatives. FRIN 48, 330–335. 10.1016/j.foodres.2012.04.009

[B42] Perkowski J. Buśko M. Stuper K. Kostecki M. Matysiak A. Szwajkowska-Michałek L. (2008). Concentration of ergosterol in small-grained naturally contaminated and inoculated cereals. Biologia (Bratisl) 63, 542–547. 10.2478/s11756-008-0083-2

[B43] Pourmirzaee T. Kelayeh S. Abedinzade M. Ghorbani A. (2019). A review on biological effects of *Lamium Album* (white dead nettle) and its components. J. Herbmed. Pharmacol. 8, 185–193. 10.15171/jhp.2019.28

[B44] Pszczółkowska A. Okorski A. Olszewski J. Jarmołkowicz J. (2013). Fungal pathogens of the genus *Fusarium* in winter wheat *Triticum Aestivum* L. protected with fungicides in north-eastern Poland. Acta Agrobot 66, 95–106. 10.5586/aa.2013.027

[B45] Riaz Ejaz M. Jaoua S. Ahmadi M. Shabani F. (2023). An examination of how climate change could affect the future spread of *fusarium* spp. around the world, using correlative models to model the changes. Environ. Technol. Innov. 31:103177. 10.1016/j.eti.2023.103177

[B46] Rys M. Saja-Garbarz D. Skoczowski A. (2022). Phytotoxic effects of selected herbal extracts on the germination, growth, and metabolism of mustard and oilseed rape. Agronomy 12:110. 10.3390/agronomy12010110

[B47] Saad A. Christopher J. Martin A. McDonald S. Percy C. (2023). Fusarium Pseudograminearum and F. culmorum affect the root system architecture of bread wheat. Crop J. 11, 316–321. 10.1016/j.cj.2022.08.013

[B48] Scherm B. Balmas V. Spanu F. Pani G. Delogu G. Pasquali M.. (2013). *Fusarium Culmorum:* causal agent of foot and root rot and head blight on wheat. Mol. Plant Pathol. 14, 323–341. 10.1111/mpp.12011 23279114 PMC6638779

[B49] Scherm B. Orrù M. Balmas V. Spanu F. Azara E. Delogu G.. (2011). Altered trichothecene biosynthesis in *TRI6*-silenced transformants of *Fusarium Culmorum* influences the severity of crown and foot rot on durum wheat seedlings. Mol. Plant Pathol. 12, 759–771. 10.1111/j.1364-3703.2011.00709.x 21726376 PMC6640217

[B50] Seepe H. A. Nxumalo W. Amoo S. O. (2021). Natural products from medicinal plants against phytopathogenic *Fusarium* species: Current research endeavors, challenges, and prospects. Molecules 26:6539. 10.3390/molecules26216539 34770948 PMC8587185

[B51] Shcherbakova L. A. Nazarova T. A. Mikityuk O. D. Istomina E. A. Odintsova T. I. (2018). An extract purified from the mycelium of a tomato wilt-controlling strain of *Fusarium Sambucinum* can protect wheat against *Fusarium* and common root rots. Pathogens 7:61. 10.3390/pathogens7030061 30011945 PMC6160971

[B52] Singh R. P. Singh P. K. Rutkoski J. Hodson D. P. He X. Jørgensen L. N.. (2016). Disease impact on wheat yield potential and prospects of genetic control. Annu. Rev. Phytopathol. 54, 303–322. 10.1146/annurev-phyto-080615-095835 27296137

[B53] Stakheev A. A. Erokhin D. V. Meleshchuk E. A. Mikityuk O. D. Statsyuk N. V. (2022). Effect of compactin on the mycotoxin production and expression of related biosynthetic and regulatory genes in toxigenic *Fusarium Culmorum*. Microorganisms 10:1347. 10.3390/microorganisms10071347 35889066 PMC9318162

[B54] Stepień Ł. Waśkiewicz A. (2013). Sequence divergence of the enniatin synthase gene in relation to the production of beauvericin and enniatins in *Fusarium* species'. Toxins 5, 537–555. 10.3390/toxins5030537 23486233 PMC3705277

[B55] Streit E. Schatzmayr G. Tassis P. Tzika E. Marin D. Taranu I.. (2012). Current situation of mycotoxin contamination and co-occurrence in animal feed focus on europe. Toxins (Basel) 4, 788–809. 10.3390/toxins4100788 23162698 PMC3496989

[B56] Sulborska A. Konarska A. Matysik-Wozniak A. Dmitruk M. Weryszko-Chmielewska E. Skalska-Kamińska A.. (2020). Phenolic Constituents of *Lamium Album* L. subsp. *Album* flowers: anatomical, histochemical, and phytochemical study. Molecules 25:6025. 10.3390/molecules25246025 33352709 PMC7766379

[B57] Sunic K. Kovac T. Loncaric A. Babic J. Sulyok M. Krska R.. (2021). *Fusarium* secondary metabolite content in naturally produced and artificially provoked fhb pressure in winter wheat. Agronomy 11, 1–19. 10.3390/agronomy11112239

[B58] Suteu D. Rusu L. Zaharia C. Badeanu M. Daraban G. M. (2020). Challenge of utilization vegetal extracts as natural plant protection products. Appl. Sci. 10:8913. 10.3390/app10248913

[B59] Tretiakova P. Voegele R. T. Soloviev A. Link T. I. (2022). Successful silencing of the mycotoxin synthesis gene *TRI5* in *Fusarium Culmorum* and observation of reduced virulence in VIGS and SIGS experiments. Genes (Basel) 13:395. 10.3390/genes13030395 35327949 PMC8953179

[B60] Urbaniak M. Waśkiewicz A. Trzebny A. Koczyk G. Stepień Ł. (2020). Cyclodepsipeptide biosynthesis in hypocreales fungi and sequence divergence of the non-ribosomal peptide synthase genes. Pathogens 9, 1–22. 10.3390/pathogens9070552 32660015 PMC7400199

[B61] Uwineza P. A. Gramza-Michałowska A. Bryła M. Waśkiewicz A. (2021). Antioxidant activity and bioactive compounds of *Lamium Album* flower extracts obtained by supercritical fluid extraction. Appl. Sci. 11:7419. 10.3390/app11167419

[B62] Uwineza P. A. Urbaniak M. Bryła M. Stepien Ł. Modrzewska M. Waśkiewicz A. (2022). *In vitro* effects of lemon balm extracts in reducing the growth and mycotoxins biosynthesis of *Fusarium Culmorum* and *F. Proliferatum*. Toxins (Basel) 14:355. 10.3390/toxins14050355 35622601 PMC9143328

[B63] Uwineza P. A. Urbaniak, M. Stepień Ł. Gramza-Michałowska A. Waśkiewicz A. (2023). *Lamium Album* flower extracts : a novel approach for controlling *Fusarium* growth and mycotoxin biosynthesis. Toxins 11:651. 10.3390/toxins15110651 37999514 PMC10675686

[B64] Veršilovskis A. Peters J. R V. D. Zomer P. Mol H. M D. N. (2019). “Biological synthesis and semi-preparative purification of zearalenone and α -zearalenol conjugates,” in EURL-MP, 1–33

[B65] Wagacha J. M. Muthomi J. W. (2007). *Fusarium Culmorum*: infection process, mechanisms of mycotoxin production and their role in pathogenesis in wheat. Crop. Prot. 26, 877–885. 10.1016/j.cropro.2006.09.003

[B66] Waśkiewicz A. Morkunas I. Bednarski W. Mai V. C. Formela M. Beszterda M. (2014). Deoxynivalenol and oxidative stress indicators in winter wheat inoculated with *Fusarium graminearum*. Toxins (Basel) 6, 575–591. 10.3390/toxins6020575 24514944 PMC3942752

[B67] Wegulo S. N. (2012).Factors influencing deoxynivalenol accumulation in small grain cereals. Toxins 4,1157–1180. 10.3390/toxins4111157 23202310 PMC3509702

[B68] Werrie P. Y. Durenne B. Delaplace P. Fauconnier M. L. (2020). Phytotoxicity of essential oils: opportunities and constraints for the development of biopesticides. A review. Foods 9, 1–24. 10.3390/foods9091291 PMC7554882 32937933

[B69] Winter M. Samuels P. L. Dong Y. Dill-macky R. (2019). Trichothecene production is detrimental to early root colonization by *Fusarium Culmorum* and *F. Graminearum* in *Fusarium* crown and root rot of wheat. Plant Pathol. 2018, 185–195. 10.1111/ppa.12929

[B70] Zhou S. Bae J. S. Bergstrom G. C. Jander G. (2018). *Fusarium Graminearum*-induced shoot elongation and root reduction in maize seedlings correlate with later seedling blight severity. Plant Direct 2:75. 10.1002/pld3.75 31245738 PMC6508817

[B71] Zubrod J. P. Bundschuh M. Arts G. Bru C. A. Kna A. Payraudeau S.. (2019). Fungicides: an overlooked pesticide class? Environ. Sci. Technol. 53:54. 10.1021/acs.est.8b04392 30835448 PMC6536136

